# Utility of cell-free DNA in diagnosing tuberculous pleurisy: A systematic review and meta-analysis protocol

**DOI:** 10.1371/journal.pone.0355485

**Published:** 2026-08-07

**Authors:** Yanqin Shen, Keying Du, Yuyang Ling, Liwei Yao

**Affiliations:** 1 Department of Nursing, Hangzhou Red Cross Hospital, Hangzhou, Zhejiang, China; 2 The Second School of Clinical Medicine, Zhejiang Chinese Medical University, Hangzhou, Zhejiang, China; Gulu University, UGANDA

## Abstract

**Background:**

Tuberculous pleurisy is the most common form of extrapulmonary tuberculosis. Diagnosis remains challenging due to the paucibacillary nature of pleural effusions, leading to low sensitivity of conventional microbiological methods and frequent reliance on invasive biopsy. Cell-free DNA (cfDNA), comprising fragmented genetic material released from host cells and pathogens into biofluids, presents a promising minimally-invasive biomarker. This protocol outlines a systematic review and meta-analysis designed to evaluate the overall diagnostic accuracy of cfDNA for tuberculous pleurisy and to identify factors influencing its performance.

**Methods:**

This protocol is prospectively registered with PROSPERO. We will systematically search PubMed, Embase, Web of Science, Scopus, The Cochrane Library from inception to June 2027. Diagnostic accuracy studies directly comparing cfDNA detection (in pleural fluid, plasma/serum) against a composite reference standard for tuberculous pleurisy (including microbiological, histological, or clinical diagnosis) will be included. Two reviewers will independently screen studies, extract data, and assess risk of bias using the QUADAS-2 tool. A bivariate random-effects meta-analysis will be performed to calculate pooled sensitivity, specificity, positive/negative likelihood ratios, and diagnostic odds ratios. A hierarchical summary receiver operating characteristic curve will be plotted. Subgroup analyses and meta-regression will explore sources of heterogeneity. The GRADE approach will be used to evaluate the certainty of evidence.

**Conclusion:**

This review will provide pooled estimates of the diagnostic sensitivity and specificity of cfDNA for tuberculous pleurisy, evaluate its clinical utility, and identify key factors—such as sample type, detection technology, and pre-analytical procedures—associated with optimal performance. The findings will offer high-level evidence to guide clinical application and future research.

**Systematic review registration:** PROSPERO Registration number: CRD420261424501

## Introduction

Tuberculous pleurisy, the most common extrapulmonary manifestation, poses a persistent diagnostic challenge [1]. Its pathogenesis involves a hypersensitivity reaction leading to lymphocyte-rich exudate and granuloma formation, which sequesters bacilli and creates a paucibacillary pleural environment [2]. This low bacterial burden undermines conventional diagnostics: culture is slow and insensitive (20–40%), smear is inadequate (<10%), and even nucleic acid amplification tests like Xpert MTB/RIF show limited sensitivity (~40%) in pleural fluid [3–6]. Consequently, diagnosis often depends on invasive biopsy [7].

Cell-free DNA (cfDNA) analysis presents a promising minimally-invasive alternative [8]. In tuberculous pleurisy, cfDNA originates both from host inflammatory cells and, more specifically, from fragmented *M. tuberculosis* genomic material [9,10]. Pathogen-derived cfDNA may be more detectable than intact bacilli in paucibacillary effusions [11]. Technologies like droplet digital PCR (ddPCR) and metagenomic next-generation sequencing (mNGS) can target this microbial cfDNA, offering potential for rapid and sensitive detection [11,12].

However, reported accuracy of cfDNA assays varies considerably [13,14], likely due to heterogeneity in sample types (pleural fluid vs. plasma), methodological protocols, and detection platforms. A definitive synthesis of evidence is lacking. This systematic review and meta-analysis therefore aims to determine the pooled diagnostic accuracy of cfDNA for tuberculous pleurisy, compare performance across sample types and technologies, and identify key sources of heterogeneity to guide future standardization and clinical application.

## Methods

### Protocol development and registration

This protocol has been designed in accordance with the Preferred Reporting Items for Systematic Review and Meta-Analysis Protocols (PRISMA-P) guidelines [15]. It has been prospectively registered on the International Prospective Register of Systematic Reviews (PROSPERO), registration number CRD420261424501. The completed systematic review will be reported following the Preferred Reporting Items for Systematic Reviews and Meta-Analyses of Diagnostic Test Accuracy (PRISMA-DTA) statement [16]. As this study involves analysis of published, publicly available data only, ethical approval from an institutional review board is not required.

### Eligibility criteria

Studies will be selected based on the PIRT framework (Participants, Index test, Reference standard, Target condition).

Participants (P): Adult or pediatric patients with clinically suspected tuberculous pleurisy, defined as presenting with pleural effusion requiring etiological investigation. No restrictions will be applied based on gender, ethnicity, or geographical location.

Index test (I): Any test detecting *M. tuberculosis* complex-specific cell-free DNA (cfDNA) as the index diagnostic method. Acceptable sample types include pleural fluid, plasma, serum, or other relevant biofluids. Detection technologies encompass all cfDNA-based molecular assays, such as qPCR, ddPCR, isothermal amplification, or mNGS targeting specific mycobacterial sequences.

Reference standard (R): A composite clinical reference standard will be used as the comparator for definitive diagnosis of tuberculous pleurisy. This standard integrates one or more of the following: Microbiological confirmation: Positive *M. tuberculosis* culture or validated NAAT (e.g., Xpert MTB/RIF Ultra) from pleural fluid, or pleural tissue. Histopathological confirmation: Demonstration of typical caseating granulomas on pleural biopsy histology, or positive acid-fast bacillus stain/culture from pleural tissue. Clinical diagnosis: A combination of consistent clinical and radiological presentation, a lymphocytic-predominant exudative effusion with elevated adenosine deaminase levels, a positive tuberculin skin test (TST) or interferon-gamma release assay (IGRA) result, and a definitive clinical and radiological response to a full course of standard anti-tuberculosis therapy.

Target condition (T): Definitively diagnosed tuberculous pleurisy.

Study Design: Cross-sectional diagnostic accuracy studies, prospective or retrospective cohort studies, and case-control studies (provided complete diagnostic 2x2 contingency table data can be extracted or derived). Studies reporting only sensitivity or specificity without sufficient data to reconstruct the 2x2 table (true positives, false positives, false negatives, true negatives) will be excluded.

### Exclusion criteria

Studies will be excluded if they meet any of the following criteria: (1) Insufficient data to populate a 2x2 contingency table, even after attempting to contact the corresponding authors; (2) Publication type is a case report, narrative review, editorial, commentary, conference abstract (without full data), protocol, or existing meta-analysis; (3) Full text is unavailable.

### Search strategy

A systematic and comprehensive search strategy will be developed and executed. The following electronic databases will be searched from inception to June 2027: PubMed, Web of Science, Embase, Scopus, and The Cochrane Library. The literature search will be conducted between 1 June and 30 June 2027. Data extraction from the included studies is scheduled for completion by 31 December 2027, and the final results are expected to be reported by 31 July 2028. The search strategy will combine controlled vocabulary (MeSH/Emtree terms) and free-text keywords centered on two core concepts: 1) tuberculous pleurisy/pleural effusion, and 2) cell-free DNA/circulating DNA. The preliminary search strategy for PubMed is illustrated below:

#1 “Tuberculosis, Pleural”[Mesh] OR “Pleural Effusion”[Mesh] OR (((tubercul* OR TB) AND (pleurisy OR pleural)) OR “pleural tuberculosis” OR “tuberculous effusion” OR “tuberculous pleurisy”)

#2 “Cell-Free Nucleic Acids”[Mesh] OR “Circulating DNA”[Mesh] OR “Cell-Free DNA” OR “cfDNA” OR “circulating DNA” OR “plasma DNA” OR “serum DNA” OR “cell-free deoxyribonucleic acid” OR “liquid biopsy” OR “cell-free Mycobacterium tuberculosis DNA” OR “cell-free MTB DNA” OR “cell-free tuberculosis DNA” OR “cell-free TB DNA” OR “TB cfDNA”

#3 #1 AND #2

No restrictions on language or publication date will be applied initially. Furthermore, the reference lists of all included studies and relevant existing systematic reviews will be manually screened to identify any additional eligible publications.

### Study selection and data extraction

Study selection will be managed using reference management software (EndNote). After duplicate removal, two independent reviewers (Yanqin Shen and Keying Du) will screen titles and abstracts, followed by a full-text assessment against the eligibility criteria. Any discrepancies will be resolved through discussion, with arbitration by a third senior reviewer (Liwei Yao) if necessary.

Data extraction will be performed independently by the same two reviewers using a pre-designed, piloted standardized form. Extracted data will include: Study Characteristics: First author, publication year, country, study design (prospective/retrospective), patient enrollment method (consecutive/random/convenience sampling). Participant Characteristics: Total sample size, age distribution (adult/pediatric), HIV co-infection status, other underlying comorbidities. Index Test and Sample Details: Specific cfDNA detection platform (e.g., ddPCR, mNGS), target gene(s) (e.g., IS6110, 16S rRNA), sample type (pleural fluid, plasma, etc.), sample processing details (centrifugation conditions, storage state, DNA extraction kit). Reference Standard Details: Specific components constituting the composite reference standard used. Diagnostic Performance Data: Raw data necessary to construct a 2x2 contingency table (counts of true positives, false positives, false negatives, true negatives).

### Risk of bias and certainty of evidence assessment

The risk of bias in included studies will be assessed using the Quality Assessment of Diagnostic Accuracy Studies-2 (QUADAS-2) tool [17]. This tool evaluates bias across four domains (patient selection, index test, reference standard, flow and timing) and applicability concerns in three domains (patient selection, index test, reference standard). Assessments will be conducted independently by two reviewers.

The certainty of the evidence for the primary outcomes (pooled sensitivity and specificity) will be evaluated using the Grading of Recommendations, Assessment, Development, and Evaluation (GRADE) approach for diagnostic tests. Evidence will be graded as high, moderate, low, or very low based on assessments of risk of bias, inconsistency, indirectness, imprecision, and publication bias.

### Data synthesis and statistical analysis

If a sufficient number of studies (≥4) are included, a bivariate random-effects meta-analysis will be conducted using Stata software (version 18.0) to pool sensitivity and specificity estimates along with their 95% confidence intervals (CIs) [18]. This model accounts for within-study variability and the negative correlation between sensitivity and specificity across studies. Positive likelihood ratio (PLR), negative likelihood ratio (NLR), and diagnostic odds ratio (DOR) with 95% CIs will also be derived. A hierarchical summary receiver operating characteristic (HSROC) curve will be plotted, and the area under the curve (AUC) will be calculated to summarize overall diagnostic performance.

Statistical heterogeneity will be investigated by visual inspection of forest plots, calculation of the I² statistic (representing the percentage of total variation across studies due to heterogeneity), and assessment of the spread of studies in the HSROC space. If substantial heterogeneity is present (I² > 50%), pre-specified subgroup analyses and random-effects meta-regression will be performed to explore potential sources. Covariates for investigation may include: sample type (pleural fluid vs. blood), cfDNA detection technology (PCR-based vs. mNGS), patient HIV status, rigor of the reference standard (microbiological/histological confirmation vs. clinical diagnosis), and study design.

Sensitivity analysis will be performed using the “leave-one-out” method to examine the influence of individual studies on the pooled estimates. If any study is found to exert a disproportionate influence on the overall results or heterogeneity, it will be reported and discussed. For any subgroup or analysis with fewer than four studies, pooled estimates will be calculated using Meta-DiSc software (version 1.4) employing a fixed-effect model with exact binomial CIs, and results will be interpreted with caution.

Given the complexities in interpreting funnel plot asymmetry in diagnostic accuracy meta-analysis, formal statistical testing for publication bias (e.g., Deeks’ funnel plot) will not be performed, but potential for publication bias will be discussed narratively [19].

## Supporting information

S1 File Preferred Reporting Items for Systematic review and Meta-Analysis Protocols (PRISMA-P) checklist. (DOCX)

S2 File Search strategies. (DOCX)
