# Supplementary material for: Utility of cell-free DNA in diagnosing tuberculous pleurisy: A systematic review and meta-analysis protocol
Source: PLoS One. 2026 Aug 7;21(8):e0355485. doi: 10.1371/journal.pone.0355485 (PMC13450758; doi:10.1371/journal.pone.0355485)
Supplement: Supplementary file 2 [file pone.0355485.s002.docx]

Search strategies

#1 "Tuberculosis, Pleural"[Mesh] OR "Pleural Effusion"[Mesh] OR (((tubercul* OR TB) AND (pleurisy OR pleural)) OR "pleural tuberculosis" OR "tuberculous effusion" OR “tuberculous pleurisy”)

#2 "Cell-Free Nucleic Acids"[Mesh] OR "Circulating DNA"[Mesh] OR "Cell-Free DNA" OR "cfDNA" OR "circulating DNA" OR "plasma DNA" OR "serum DNA" OR “cell-free deoxyribonucleic acid” OR "liquid biopsy" OR “cell-free Mycobacterium tuberculosis DNA” OR “cell-free MTB DNA” OR “cell-free tuberculosis DNA” OR “cell-free TB DNA” OR “TB cfDNA”

#3 #1 AND #2
